# Reduced osteoprotegerin expression by osteocytes may contribute to rebound resorption after denosumab discontinuation

**DOI:** 10.1172/jci.insight.167790

**Published:** 2023-09-22

**Authors:** Qiang Fu, Nancy C. Bustamante-Gomez, Humberto Reyes-Pardo, Igor Gubrij, Diana Escalona-Vargas, Jeff D. Thostenson, Michela Palmieri, Joseph J. Goellner, Intawat Nookaew, C. Lowry Barnes, Jeffrey B. Stambough, Elena Ambrogini, Charles A. O’Brien

**Affiliations:** 1 Center for Musculoskeletal Disease Research,; 2 Division of Endocrinology and Metabolism,; 3 Department of Pediatrics,; 4 Department of Biostatistics,; 5 Department of Biomedical Informatics, and; 6 Department of Orthopaedic Surgery, University of Arkansas for Medical Sciences, Little Rock, Arkansas, USA.; 7 Central Arkansas Veterans Healthcare System, Little Rock, Arkansas, USA.

**Keywords:** Bone Biology, Osteoclast/osteoblast biology

## Abstract

Denosumab is an anti-RANKL Ab that potently suppresses bone resorption, increases bone mass, and reduces fracture risk. Discontinuation of denosumab causes rapid rebound bone resorption and bone loss, but the molecular mechanisms are unclear. We generated humanized RANKL mice and treated them with denosumab to examine the cellular and molecular conditions associated with rebound resorption. Denosumab potently suppressed both osteoclast and osteoblast numbers in cancellous bone in humanized RANKL mice. The decrease in osteoclast number was not associated with changes in osteoclast progenitors in bone marrow. Long-term, but not short-term, denosumab administration reduced osteoprotegerin (OPG) mRNA in bone. Localization of OPG expression revealed that OPG mRNA is produced by a subpopulation of osteocytes. Long-term denosumab administration reduced osteocyte OPG mRNA, suggesting that OPG expression declines as osteocytes age. Consistent with this, osteocyte expression of OPG was more prevalent near the surface of cortical bone in humans and mice. These results suggest that new osteocytes are an important source of OPG in remodeling bone and that suppression of remodeling reduces OPG abundance by reducing new osteocyte formation. The lack of new osteocytes and the OPG they produce may contribute to rebound resorption after denosumab discontinuation.

## Graphical abstract

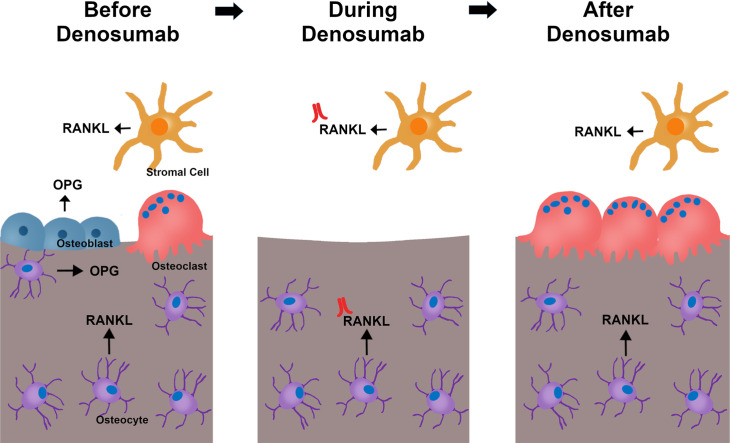

## Introduction

Fractures and disability caused by osteoporosis are major health concerns worldwide (1). The low bone mass that is the basis for osteoporosis is often caused by excessive bone resorption by osteoclasts. Receptor activator of NF-κB ligand (RANKL) is a cytokine that stimulates osteoclast differentiation, activity, and survival (2), and osteoprotegerin (OPG) is a naturally occurring antagonist of RANKL (3). Denosumab is an anti-RANKL mAb that potently inhibits bone resorption (4, 5). Administration of denosumab once every 6 months to postmenopausal women increases bone mass and decreases fracture risk (6–8). Despite its efficacy, discontinuation of denosumab results in a rapid increase in bone resorption markers as early as 9 months after the last injection and bone loss within 12 months after the last injection (7, 9). Importantly, the increase in resorption exceeds pretreatment levels and, 1 year after discontinuation, bone mass can return to, or be lower than, pretreatment levels (7, 9). Although transition to other antiresorptive therapies can blunt the bone loss after denosumab discontinuation, this is not always effective or possible (10). Thus, the rapid increase in resorption, which has been referred to as “rebound resorption” (11), is a clinically important but poorly understood cause of bone loss and fractures.

The mechanisms responsible for rebound resorption after denosumab discontinuation are unknown. A 2016 editorial suggested that rebound resorption “could be due to an expanded pool of osteoclast precursors” or “a high RANKL/OPG ratio” (10). Osteocytes, which are former osteoblasts buried in bone matrix, can control both bone resorption and bone formation (12). An increased number of empty osteocyte lacunae has been observed in patients after denosumab discontinuation, leading to the suggestion that accumulation of dead osteocytes might drive rebound resorption (13). In addition, a recent study in mice, in which recombinant OPG was used to suppress resorption, identified a novel cell type, termed an osteomorph, produced by the fission of osteoclasts. The authors suggested that accumulation of these cells may contribute to rebound resorption (14).

We sought to identify possible mechanisms underlying rebound resorption, using mice. Because denosumab does not bind murine RANKL (5), we modified the endogenous murine *Tnfsf11* gene, which encodes RANKL, to produce a version of RANKL that is inhibited by denosumab. Administration of denosumab to these mice potently suppressed resorption, which rebounded above vehicle-treated levels after discontinuation. Denosumab potently suppressed both osteoclast and osteoblast numbers after several weeks of administration, and this was associated with reduced expression of *Tnfrsf11b*, which encodes OPG, in bone. High-resolution analysis using ISH demonstrated that osteoblasts and osteocytes express *Tnfrsf11b* and that denosumab eliminated osteoblasts and reduced expression of *Tnfrsf11b* by osteocytes. We did not observe changes in osteoclast progenitors in the bone marrow of denosumab-treated mice. Together, these results suggest that loss of OPG in the bone microenvironment helps set the stage for rebound resorption.

## Results

### Development of humanized RANKL mice.

**Figure 1:**
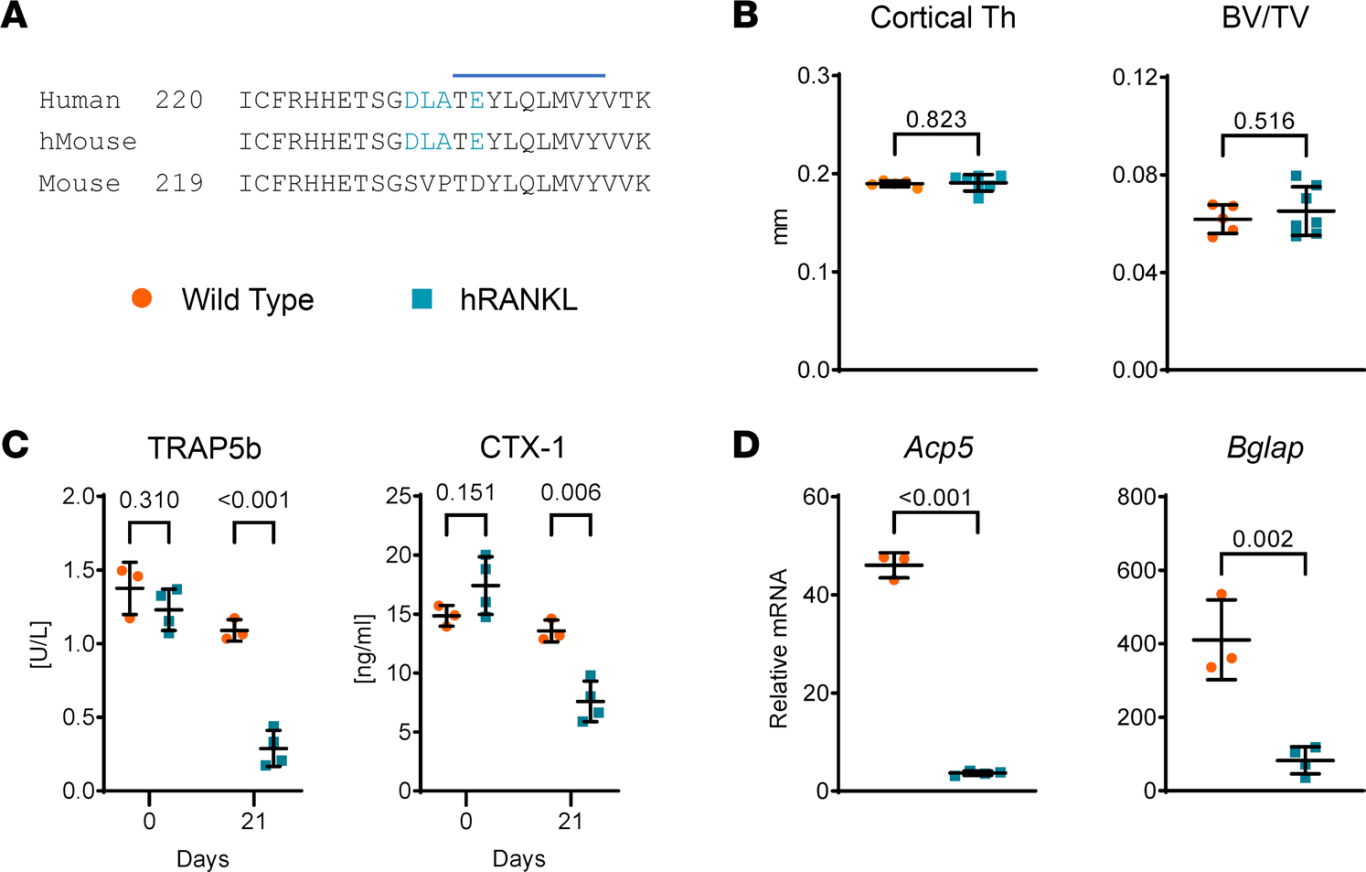
Creation of hRANKL mice. (**A**) Amino acid sequence comparison of the region of the RANKL protein harboring the 9 amino acid epitope bound by denosumab, identified by Schieferdecker et al. (15). The epitope is indicated by the horizontal blue line. The 4 amino acids encoded by the murine gene that were modified to match the human sequence are shown in light blue. (**B**) Femoral cortical thickness (Cortical Th) and cancellous bone volume (BV/TV) of untreated, 12-week-old, female WT and hRANKL mice. *P* values determined by 2-tailed, unpaired *t* test. WT mice, *n* = 5; hRANKL mice, *n* = 7. (**C**) Four-month-old male WT or homozygous hRANKL mice were injected once per week with denosumab at a dose of 10 mg/kg BW for 3 weeks. Blood was collected on the day of the first injection and 1 week after the last injection, and serum TRAP5b and CTX-1 were measured by ELISA. *P* values determined by 2-tailed, unpaired *t* test and Holm-Šídák adjustment for multiple comparisons. WT mice, *n* = 3, hRANKL mice, *n* = 4. (**D**) Gene expression in cortical bone of the same mice used in **C**. *P* values determined by 2-tailed, unpaired *t* test. Bars and whiskers represent the mean ± SD.

We sought to develop a murine model of rebound resorption to study its molecular causes. Although recombinant OPG effectively suppresses bone resorption in mice, the cost of this reagent would be prohibitive for repeated in vivo experiments (5). A version of humanized RANKL (hRANKL) mice was previously developed by the pharmaceutical company Amgen (5). However, because these mice are not widely available, we sought to create a new version of hRANKL mice that would allow use of denosumab purchased from a clinical pharmacy to suppress bone resorption in mice. The hRANKL mice created by Amgen were generated by replacing the entirety of *Tnfsf11* exon 5 with human exon 5 (5). Because the epitope bound by denosumab has subsequently been identified (15), we used this information and CRISPR-based gene editing to alter only the 4 amino acids necessary to introduce the human epitope into the endogenous *Tnfsf11* gene (Figure 1A).

Analysis of cortical thickness and cancellous bone volume of untreated mice by μCT demonstrated that the 4 amino acid changes in hRANKL mice did not alter bone mass compared with WT mice (Figure 1B). Administration of denosumab once per week for 3 weeks to mice homozygous for the humanized *Tnfsf11* allele potently suppressed bone resorption, as measured by the serum resorption markers TRAP5b and CTX-1, but had no effect on these markers in WT mice (Figure 1C). Consistent with this, *Acp5* mRNA, which encodes the osteoclast marker enzyme tartrate-resistant acid phosphatase (TRAP), was reduced in the bones of hRANKL mice compared with WT controls after 21 days of denosumab treatment (Figure 1D). The mRNA encoding the osteoblast product osteocalcin (*Bglap*) was also reduced by denosumab in hRANKL mice, consistent with the coupling of bone formation to bone resorption (Figure 1D).

### Discontinuation leads to rebound resorption.

**Figure 2:**
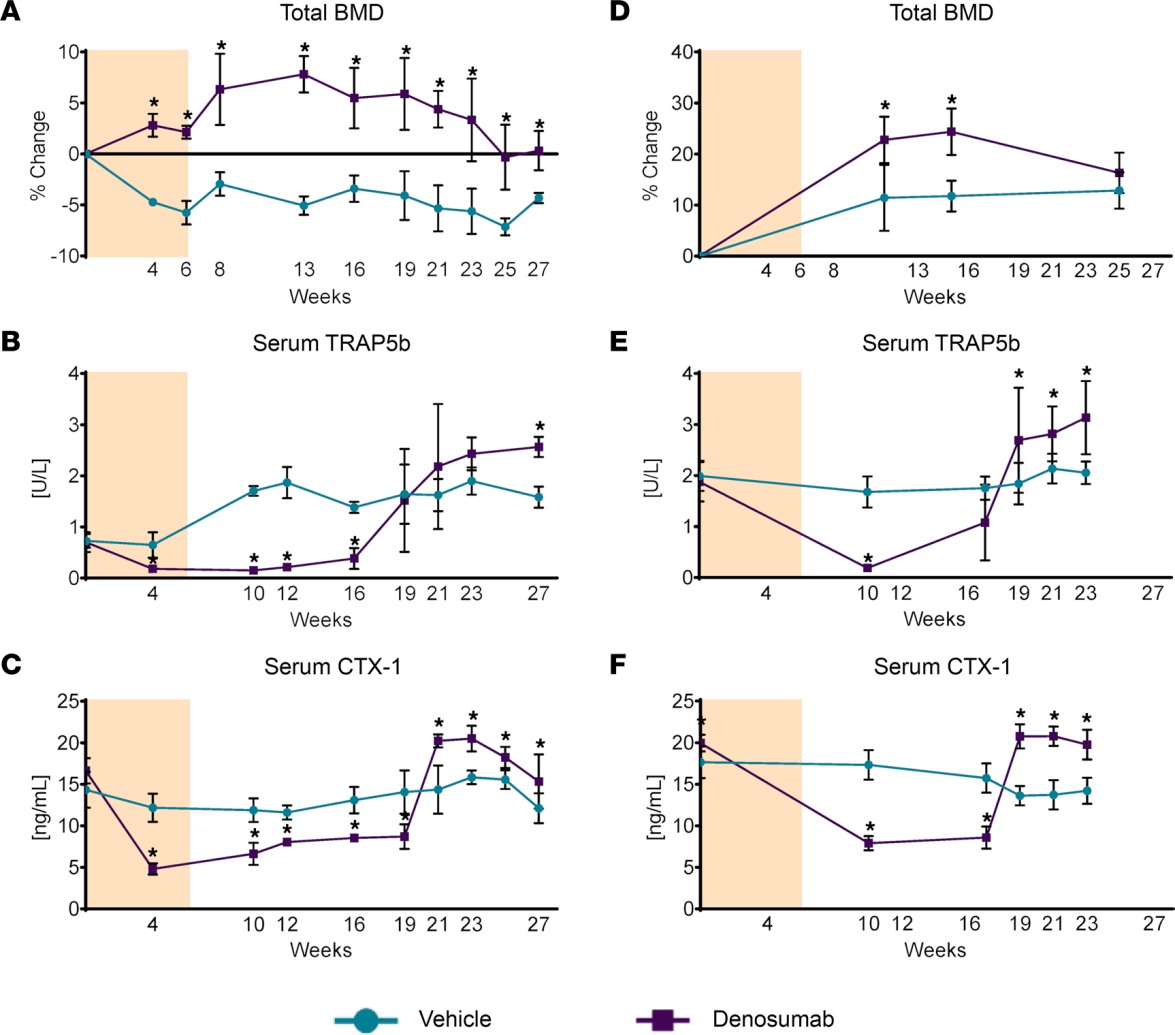
Rebound resorption in hRANKL mice. (**A**) Six-month-old, male hRANKL mice were injected with vehicle or denosumab (10 mg/kg) once every 2 weeks for a total of 4 doses (tan-shaded area) and BMD was measured by dual-energy x-ray absorptiometry at the indicated time points. (**B** and **C**) TRAP5b or CTX-1 levels were measured in serum of the same mice as in **A** by ELISA at the indicated time points. *n* = 3 mice/group. Asterisks indicate *P* value ≤ 0.02 by mixed-effects repeated measures model. (**D**) Serial BMD analysis of 10-week-old, female hRANKL mice treated as in **A**. (**E** and **F**) Serum TRAP5b or CTX-1 levels of the same mice as in **D**. *P ≤ 0.02 by mixed effects repeated measures model. Vehicle-treated mice, *n* = 6; denosumab-treated mice, *n* = 8, except for the 23 day time point, at which *n* = 7 mice due to depletion of 1 sample. All values are the mean ± SD.

We next determined whether discontinuation of denosumab administration would lead to rebound resorption. To do this, 6-month-old male mice received vehicle or denosumab every 2 weeks for a total of 4 doses (Figure 2A). Denosumab increased bone mineral density (BMD) above that of vehicle-treated controls by week 4, and this difference was maintained until week 27 (Figure 2A). Serum TRAP5b was reduced by week 4 in the denosumab group but returned to vehicle-treated levels by week 19 (Figure 2B). By week 27, the levels of TRAP5b in the denosumab-treated mice exceeded those in the vehicle-treated mice, demonstrating a rebound phenomenon similar to that observed in patients after discontinuation of denosumab (Figure 2B). A similar pattern of rebound resorption was observed when we measured CTX-1 (Figure 2C). We also administered denosumab or vehicle to 10-week-old female mice using the same dosing regimen and again observed rebound resorption with similar timing (Figure 2, D–F).

### Denosumab potently suppresses bone remodeling.

**Figure 3:**
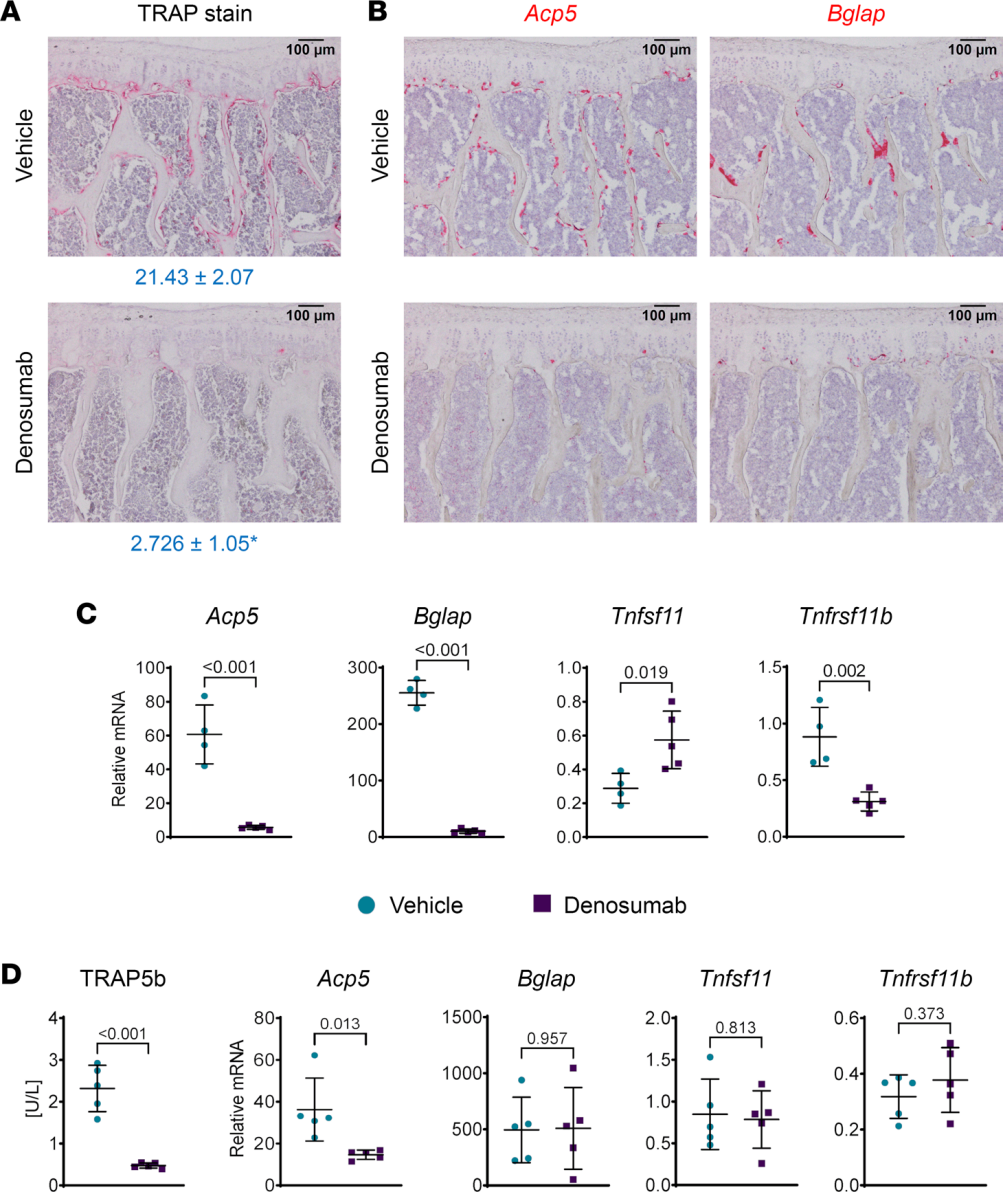
Denosumab potently suppresses remodeling in hRANKL mice. Ten-month-old, male hRANKL mice were injected with vehicle or denosumab (10 mg/kg) once every 2 weeks for a total of 4 doses. Bones were harvested 2 weeks after the last dose. (**A**) TRAPase stain of vertebral bone sections from vehicle- or denosumab-treated mice. The osteoclast surface per bone surface is shown below the images. *P < 0.01 by 2-tailed, unpaired *t* test. *n* = 3 per group. (**B**) ISH with probes for *Acp5* or *Bglap* using vertebral bone sections from vehicle- or denosumab-treated mice. (**C**) Gene expression of the indicated genes using TaqMan assays of RNA from cortical bone. Data are shown as mean ± SD and *P* values are from 2-tailed, unpaired *t* tests. Vehicle-treated mice, *n* = 4; denosumab-treated mice, *n* = 5. (**D**) Three-month-old male mice were treated with a single dose of vehicle or denosumab (10 mg/kg) and tissues harvested 48 hours after injection. Serum levels of TRAP5b were measured by ELISA and gene expression of the indicated transcripts was measured in RNA from femoral cortical bone by TaqMan assays. Data are shown as mean ± SD and *P* values are from 2-tailed, unpaired *t* tests. *n* = 5 per group. Scale bars: 100 µm.

To identify cellular and molecular conditions that may set the stage for rebound resorption, we injected 10-month-old male mice with vehicle or denosumab every 2 weeks for a total of 4 doses and collected bones for analysis 2 weeks after the last dose. Two weeks after the last dose, which was 8 weeks after the first dose, osteoclasts were greatly reduced in cancellous bone of mice receiving denosumab (Figure 3A). ISH, using a probe for *Acp5*, confirmed the results obtained using TRAP histostaining (Figure 3B). Hybridization using a *Bglap* probe revealed an almost complete absence of osteoblasts as well (Figure 3B), which is consistent with results in humans receiving long-term denosumab administration (16) and with the requirement of prior bone resorption for osteoblast formation in remodeling bone (17). Similar results were obtained in a second experiment in which mice received 1 dose fewer of denosumab and were analyzed by standard histomorphometry (Supplemental Figure 1; supplemental material available online with this article; https://doi.org/10.1172/jci.insight.167790DS1). The profound decrease in the bone formation rate in this second experiment reflects the almost complete absence of osteoblasts in the denosumab-treated mice (Supplemental Figure 1).

The lack of osteoclasts and osteoblasts was also reflected in gene expression analysis, which showed much lower *Acp5* and *Bglap* mRNA levels in cortical bone from the denosumab-treated mice (Figure 3C). To determine whether changes in the RANKL/OPG ratio occur under these conditions, we quantified *Tnfsf11* and *Tnfrsf11b* mRNA in the same bones and found that denosumab increased *Tnfsf11* mRNA and reduced *Tnfrsf11b* mRNA (Figure 3C). Similar results were obtained in a second experiment except that *Tnfsf11* mRNA levels were unchanged by denosumab (Supplemental Figure 1).

Previous studies have shown that osteoclast number declines rapidly after administration of OPG to mice (18). To determine if similar events occur in hRANKL mice after denosumab administration and whether OPG levels are altered by acute reductions in osteoclasts, we injected mice with a single dose of denosumab and collected serum and bones 48 hours after injection. The serum level of TRAP5b was much lower in the denosumab-treated mice and this was associated with lower *Acp5* mRNA in cortical bone (Figure 3D). In contrast, there were no changes in *Bglap, Tnfsf11*, or *Tnfrsf11b* 48 hours after denosumab administration (Figure 3D).

### High OPG production by new osteocytes.

**Figure 4:**
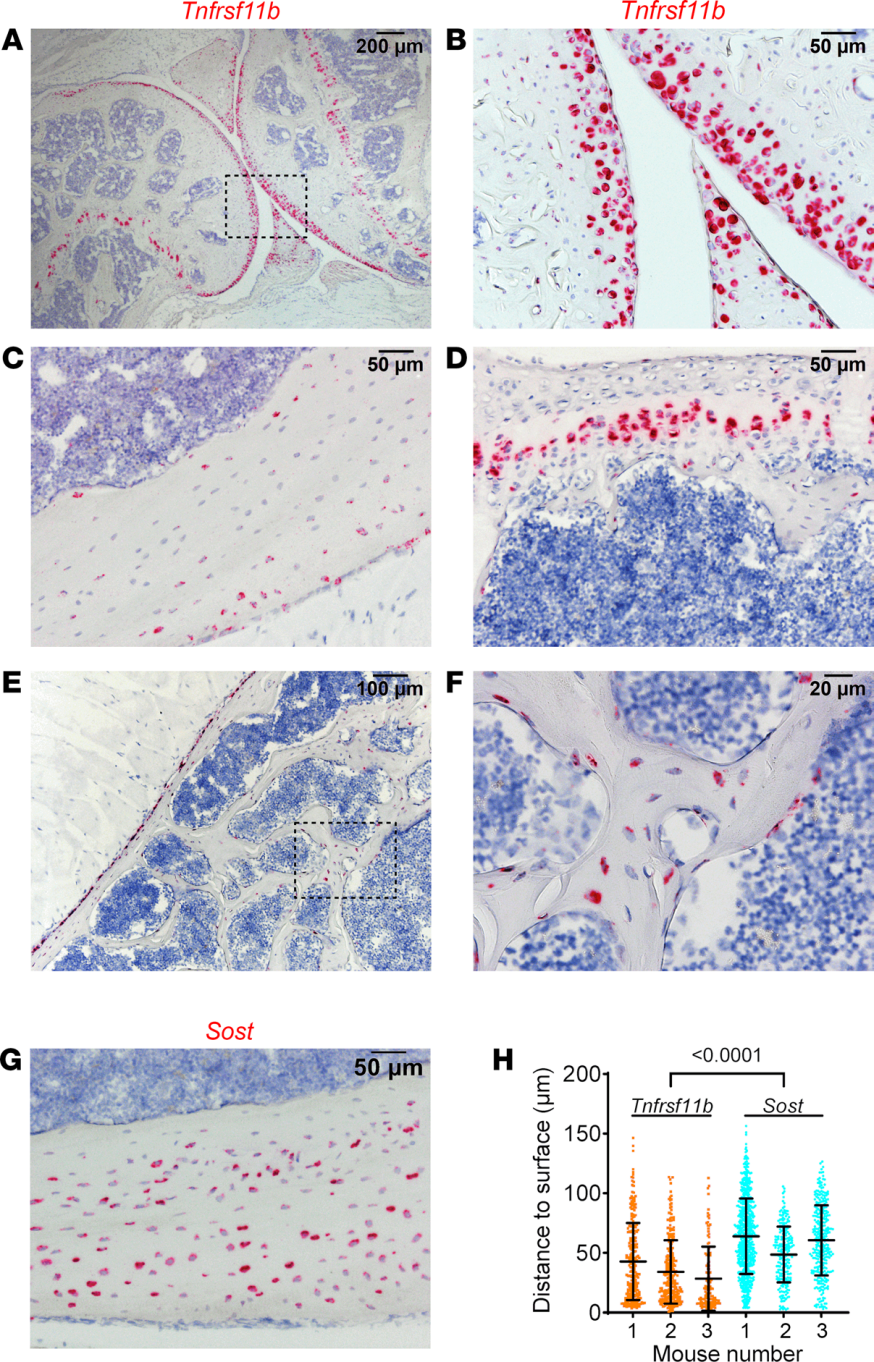
Chondrocytes and osteocytes express *Tnfrsf11b*. All results in this figure are from 5-month-old male mice. (**A**–**F)** ISH performed with a probe for *Tnfrsf11b*. (**A**–**C**) Knee joint and femoral cortical bone. **B** is a higher-powered magnification of the boxed region in **A**. (**D**–**F**) Vertebral bone. **F** is a higher-powered magnification of the boxed region in **E**. **G** shows ISH performed with a probe for *Sost*. (**H**) Distance of *Tnfrsf11b*^+^ or *Sost*^+^ osteocytes from the nearest femoral cortical surface. Data are shown as mean ± SD and the *P* value is from a nested ANOVA model. Osteocytes for each gene were measured in 3 mice. Scale bars: 200 µm (**A**), 100 µm (**E**), 50 µm (**B**–**D**, **G**), 20 µm (**F**).

Because the changes in *Tnfrsf11b* were larger and more consistent than with *Tnfsf11*, we sought to identify the basis for the reduction in *Tnfrsf11b* by denosumab. To accomplish this, we first performed ISH using RNAscope on bones from untreated mice to identify which cell types express *Tnfrsf11b*. Femur sections hybridized with a *Tnfrs11b* probe revealed that this mRNA is produced by a variety of cell types, including articular chondrocytes, a discrete population of chondrocytes in the growth plate, and osteocytes (Figure 4, A and B). In femoral cortical bone, the probe labeled osteocytes as well as periosteal cells with a location and morphology consistent with osteoblasts (Figure 4C). Hybridization of vertebral bone sections revealed expression in growth-plate chondrocytes, periosteal cells, and in osteocytes (Figure 4, D–F).

The pattern of *Tnfrsf11b*-expressing osteocytes in cortical bone suggested that the highest-expressing osteocytes were near the bone surface. To determine if this was the case, we quantified the distance of *Tnfrsf11b*-expressing osteocytes from the nearest cortical surface and compared that with the distance of *Sost*-expressing osteocytes from the bone surface (Figure 4, G and H). *Tnfrsf11b*-expressing osteocytes, on average, were significantly closer to the bone surface than were *Sost*-expressing osteocytes. One possible explanation for this pattern of expression is that newly formed osteocytes express higher levels of *Tnfrsf11b* than older osteocytes.

**Figure 5:**
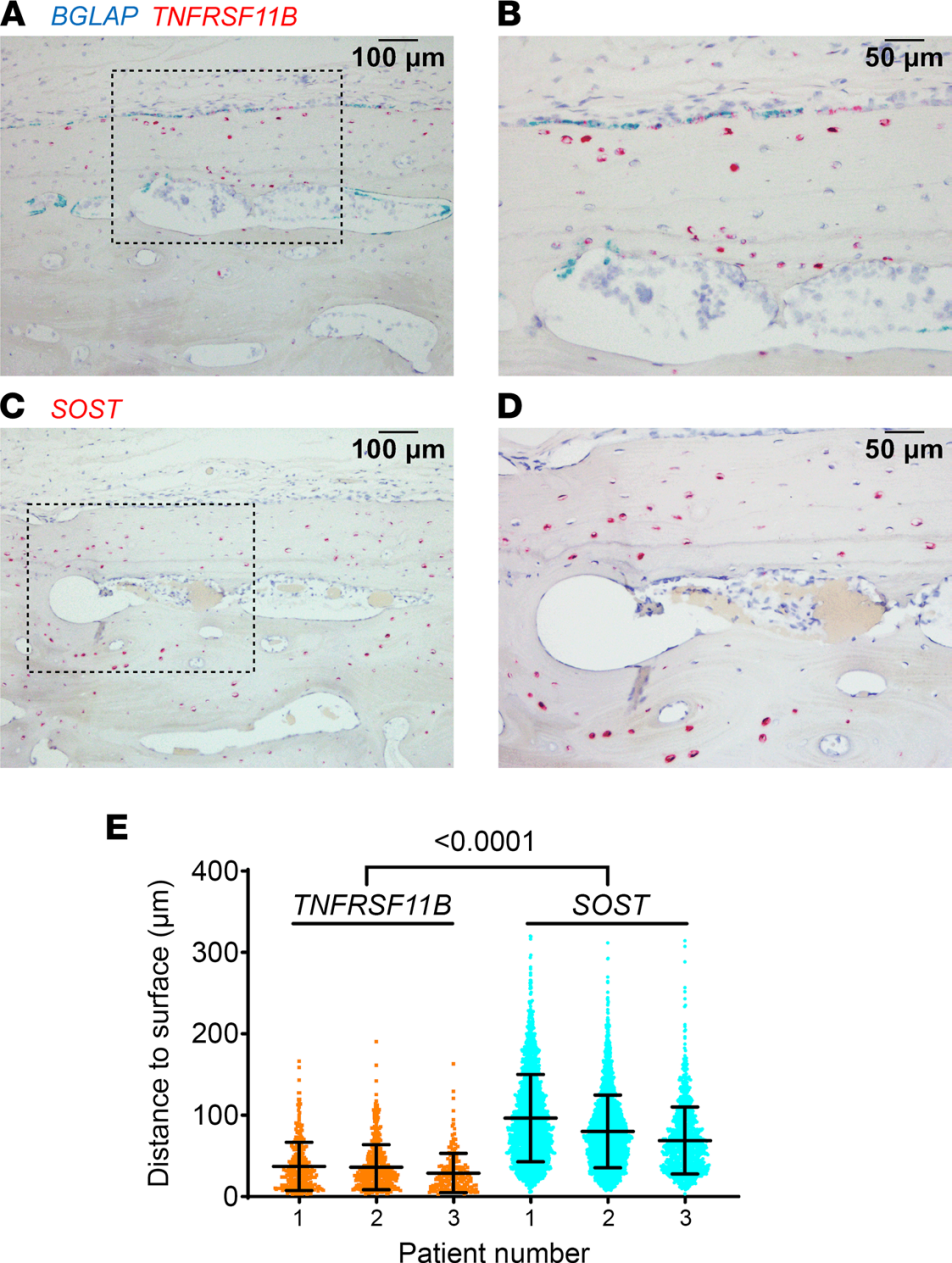
Human osteocytes expressing *TNFRSF11B* reside near the bone surface. (**A** and **B**) ISH of human femoral cortical bone, using a probe for *BGLAP* (blue) and *TNFRSF11B* (red), from a 48-year-old male patient. **B** shows a higher magnification image of the boxed region in **A**. (**C** and **D**) ISH of a serial section from the same bone used in **A** and **B** using a probe for *SOST* (red). **D** shows a higher magnification image of the boxed region in **C**. (**E**) Distance of *TNFRSF11B*^+^ or *SOST*^+^ osteocytes from the nearest bone surface. Data are shown as mean ± SD and the *P* value is from a nested ANOVA model. Osteocytes for each gene were measured in 3 patients. Scale bars: 100 µm (**A** and **C**), 50 µm (**B** and **D**).

To determine if a similar pattern of expression exists in humans, we detected *TNFRSF11B* mRNA in sections of femoral cortical bone. In these samples, *TNFRSF11B* was expressed by osteoblasts on the bone surface that also expressed *BGLAP* as well as a subpopulation of osteocytes that appeared to be predominantly near the periosteal surface or near Haversian canals (Figure 5, A and B). Quantification of the distance to the nearest bone surface (either the periosteal surface or a Haversian canal) revealed that *TNFRSF11B*-expressing osteocytes were significantly closer to such surfaces than were *SOST*-expressing osteocytes (Figure 5, C–E).

**Figure 6:**
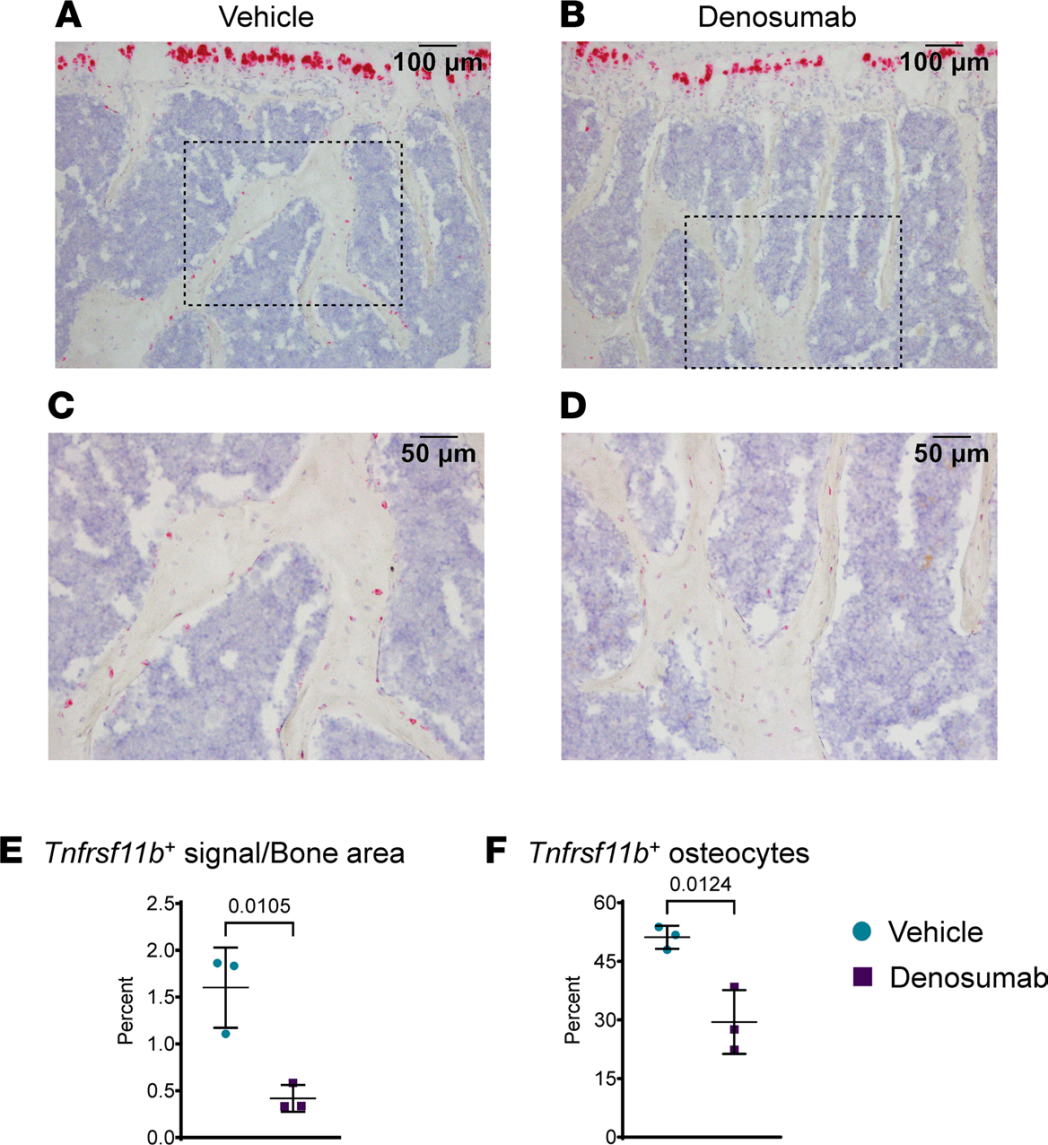
Denosumab suppresses *Tnfrsf11b* expression in osteocytes. Ten-month-old, male hRANKL mice were injected with vehicle or denosumab (10 mg/kg) once every 2 weeks for a total of 4 doses, and bones were harvested 2 weeks after the last injection. (**A**–**D**) ISH with a probe for *Tnfrsf11b* (red) was performed on vertebral bone sections. **C** and **D** show higher magnification images of the boxed regions in **A** and **B**, respectively. (**E**) Quantification of the red signal area/cancellous bone area. (**F**) Quantification of the number of *Tnfrsf11b*^+^ osteocytes in cancellous bone. Data are shown as mean ± SD, and the *P* values are from a 2-tailed, unpaired *t* test. *n* = 3 per group. Scale bars: 100 µm (**A** and **B**), 50 µm (**C** and **D**).

We then examined the impact of denosumab on the pattern of *Tnfrsf11b* expression using bones from mice injected once every 2 weeks for 4 doses and then harvested 2 weeks after the last dose. Denosumab did not have an obvious impact on *Tnfrsf11b* expression in growth-plate chondrocytes but reduced expression in osteocytes (Figure 6, A–D). We quantified the change in osteocyte *Tnfrsf11b* expression in 2 ways. First, we quantified the intensity of the hybridization signal in osteocytes using image analysis and confirmed an approximately 4-fold reduction in the intensity of signal in cancellous bone (Figure 6E). Second, we quantified the number of osteocytes expressing *Tnfrsf11b* and found that this was reduced by about half (Figure 6F). The reduction in osteocyte *Tnfrsf11b* with denosumab is consistent with the idea that this gene may be predominantly expressed by newly formed osteocytes and that its expression declines upon osteocyte maturation.

We have previously reported that osteoblasts, but not osteocytes, are a major source of OPG in remodeling cancellous bone (19). This conclusion was based on a much greater reduction in bone mass in mice lacking OPG in cells targeted by a Dmp1-Cre transgene compared with mice lacking OPG in cells targeted by a Sost-Cre transgene. Because Dmp1-Cre targets osteoblasts and osteocytes, whereas Sost-Cre targets osteocytes but not osteoblasts, we attributed the high resorption in the Dmp1-Cre mice to loss of OPG in osteoblasts but not osteocytes. This conclusion is not consistent with high expression of *Tnfrsf11b* by osteocytes in our present study. A possible explanation for the mild phenotype of mice lacking OPG in Sost-Cre–targeted cells is that this Cre driver strain does not efficiently target newly formed osteocytes, which appear to produce more OPG than do osteoblasts or more mature osteocytes. To address this possibility, we re-examined bone sections from mice in which a Cre reporter gene was activated by the Sost-Cre transgene or a Dmp1-Cre transgene. Although Dmp1-Cre targeted virtually all osteocytes, Sost-Cre targeted about 20% less (Supplemental Figure 2) and the nontargeted osteocytes were primarily located near the periosteal or endosteal surface (Supplemental Figure 2). This pattern of nontargeted osteocytes is similar to the pattern of *Tnfrsf11b*-expressing osteocytes. These findings suggest that Sost-Cre does not target new osteocytes and that the low bone mass we observed in Dmp1-Cre;Tnfrsf11b-fl/fl mice was due to loss of *Tnfrsf11b* in new osteocytes and perhaps osteoblasts, some of which also express *Tnfrsf11b* (Supplemental Figure 3).

### Denosumab does not increase osteoclast progenitors.

**Figure 7:**
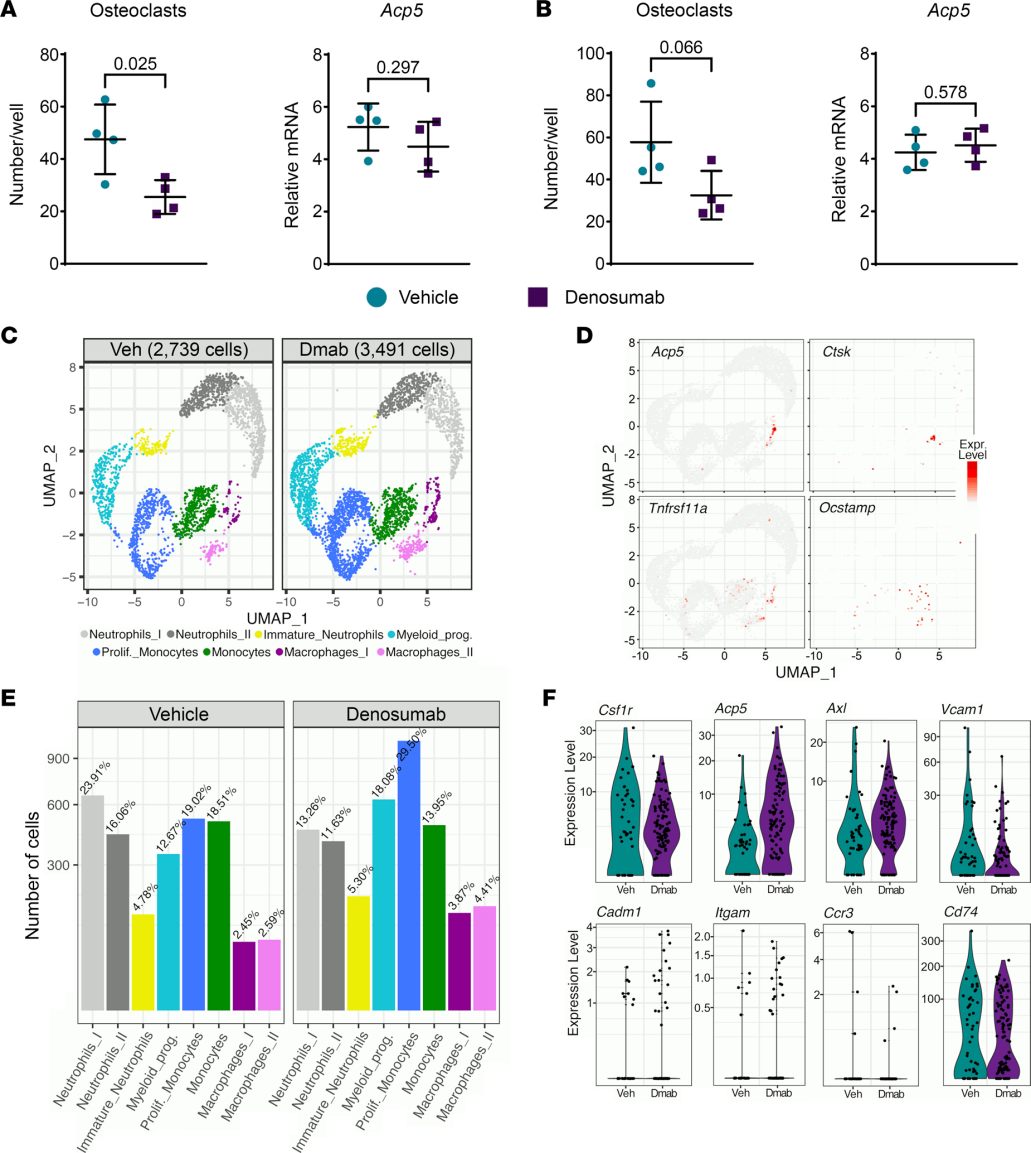
Denosumab does not increase osteoclast progenitors. (**A**) Left panel: In vitro osteoclast formation using bone marrow from 7-month-old female mice treated with vehicle (Veh) or denosumab (Dmab; 10 mg/kg every 2 weeks). Bone marrow was isolated 2 weeks after the last of 4 injections. Bone marrow from each mouse was cultured in 3 wells and each symbol represents the average osteoclast number of triplicate wells. Right panel: *Acp5* mRNA levels in RNA from freshly isolated bone marrow of the same mice described in the left panel. In both panels, bars and whiskers represent the mean ± SD of 4 mice and *P* values were determined by 2-tailed unpaired *t* test. (**B**) As in **A,** except 10-month-old male mice were used. (**C**) UMAPs from scRNA-Seq analysis of Cd11b^+^ bone marrow cells isolated from 13-month-old male mice treated the same as in **A**. (**D**) UMAPs showing expression of genes highly expressed in osteoclasts. Each panel contains data from vehicle- and denosumab-treated mice. (**E**) Comparison of relative cell numbers from each cluster shown in **C**. (**F**) Violin plots of cells in the Macrophage_I cluster for genes highly expressed in osteomorphs. prolif., proliferative; prog., progenitors.

Accumulation of osteoclast progenitors has been proposed as an explanation for rebound resorption (10, 14). To determine whether osteoclast progenitors accumulate in hRANKL mice after denosumab administration, we performed osteoclastogenesis assays in vitro using bone marrow harvested from mice 2 weeks after the last injection of vehicle or denosumab (4 doses total, marrow harvested 8 weeks after the first dose). Rather than an increase, we observed a slight decrease in osteoclast formation in mice treated with denosumab compared with control mice (Figure 7A). In this same experiment, we also quantified *Acp5* mRNA in freshly isolated bone marrow cells based on the demonstration that osteomorphs, which may serve as a source of osteoclasts, express high levels of this osteoclast marker gene (14). However, the levels of *Acp5* were not different between the treatments (Figure 7A). A repeat of this experiment yielded similar results (Figure 7B). These results are consistent with previous studies showing that the osteoclastogenic potential of bone marrow was not increased by administration of OPG to mice (18).

We also used single-cell RNA-Seq (scRNA-Seq) in an attempt to identify cells with a gene expression profile consistent with osteomorphs and determine whether the abundance of these cells changed with denosumab administration. Because osteomorphs are reported to express high levels of Cd11b, encoded by *Itgam* (14), we isolated Cd11b^+^ cells from the bones of vehicle- and denosumab-treated mice 2 weeks after the last injection of denosumab (4 doses total). FACS-isolated cells were subjected to scRNA-Seq using the 10X Chromium platform. We obtained known myeloid cell types with both treatments, including neutrophils, myeloid progenitors, monocytes, and macrophages, but there were no obvious differences in the number of clusters from mice treated with vehicle versus denosumab (Figure 7C). Although the Macrophage_I cluster harbored cells expressing some osteoclast marker genes, these cells did not express *Ocstamp* and thus do not appear to represent mature osteoclasts (Figure 7D). The reason for the lack of mature osteoclasts is unclear but may result from an inability of mature osteoclasts to survive the isolation protocol or the inability of the 10X Chromium Controller to sort large, multinucleated cells.

To determine whether cells consistent with osteomorphs were present in Cd11b^+^ cells from either treatment, we compared expression levels of 6 genes proposed as cell surface markers for osteomorphs (14). The Macrophage_I cluster had the profile most consistent with that proposed for osteomorphs (Supplemental Figure 4), but the relative numbers of these cells did not differ greatly between vehicle- and denosumab-treated mice (Figure 7E). Nonetheless, there was a trend toward more cells expressing some of the osteomorph markers in the denosumab-treated mice (Figure 7F).

## Discussion

There are several potential explanations for rebound resorption after denosumab discontinuation. Osteoclast progenitors may accumulate and then rapidly differentiate into osteoclasts after discontinuation. Production of RANKL and OPG may be altered such that excessive resorption occurs. Accumulation of osteocyte death may stimulate excessive resorption. Last, whatever molecular components constitute the mechanostat may sense an excess of bone relative to biomechanical need and send signals to reduce bone mass by stimulating resorption (20). These potential explanations are not mutually exclusive and could even be related. For example, the mechanostat might direct changes in the RANKL/OPG ratio.

Presently, there is no functional evidence, to our knowledge, that supports any of these or other mechanisms. In other words, no one has inhibited 1 of these mechanisms and examined the impact on rebound resorption. A recent study presented evidence that blockade of RANKL in mice using OPG-Fc results in fission of osteoclasts into smaller cells that retain a gene expression profile similar to osteoclasts, referred to osteomorphs (14). On the basis of this finding, the authors postulated that these committed cells might remain in the bone microenvironment and rapidly re-fuse into multinucleated osteoclasts after denosumab discontinuation. However, we did not observe accumulation of osteoclast progenitors in the bone marrow microenvironment after denosumab administration, nor did we detect a clear accumulation of cells with an expression profile consistent with osteomorphs.

In contrast, we provide multiple lines of evidence that the amount of OPG in the bone microenvironment is reduced by prolonged administration of denosumab and that this is associated with the loss of osteoblasts and newly formed osteocytes. Our ISH results suggest that newly formed osteocytes are the predominant source of OPG in bone. These results are consistent with studies by Kramer et al. (21) showing that osteocytes produce more OPG than do osteoblasts. Articular chondrocytes and a population of growth-plate chondrocytes also express high levels of *Tnfrsf11b*. However, it is unlikely that chondrocytes contribute to suppression of resorption, because circulating OPG, at least at physiological levels, does not inhibit bone resorption (19, 22). The role of chondrocyte OPG is unclear, but expression in the growth plate may block resorption of the growth plate by the robust osteoclast formation present in the primary spongiosa (23, 24).

We previously reported that osteoblasts, but not osteocytes, are the major source of OPG in cancellous bone (19). In that study, we did not recognize that the Sost-Cre transgene we used does not efficiently target newly formed osteocytes. Considering this new information, and the results of the ISH presented herein, the low bone mass in mice lacking *Tnfrsf11b* in Dmp1-Cre–targeted cells is best explained by loss of OPG in newly formed osteocytes with, perhaps, some contribution by the loss in osteoblasts.

The loss of OPG in the bone microenvironment provides a compelling explanation for rebound resorption. Specifically, the sequence of events leading to rebound may occur as follows. Prior to denosumab administration, the actions of RANKL are opposed by OPG produced by newly formed osteocytes. Denosumab administration results in a rapid loss of osteoclasts and the subsequent loss of osteoblasts due to the loss of coupling factors. Over time, the number of newly formed osteocytes also declines as bone formation declines. During continued denosumab administration, bone turnover remains low and bone mass remains stable or increases. Upon discontinuation, denosumab levels in the circulation decline and eventually reach the threshold below which RANKL, produced by stromal cells and osteocytes, is no longer inhibited. However, because the levels of OPG are lower than they were prior to administration, and because RANKL abundance is either unchanged or possibly elevated, the magnitude of the pro-osteoclastogenic signal is much greater than before treatment and will remain so until osteoblast and new osteocyte formation restore OPG abundance. This scenario also provides an explanation for the transient nature of rebound resorption.

Rebound resorption does not occur after discontinuation of bisphosphonates (25). Because bisphosphonates bind to the bone matrix, they are retained in the body long after administration. This results in a slow release from the skeleton, which is associated with a slow increase in resorption markers (10). This slow return to bone remodeling may allow accumulation of new osteocytes and their OPG at a rate that is sufficient to prevent rebound resorption. In contrast, once the levels of denosumab fall below a threshold level, it is likely that resorption rapidly resumes in all remodeling bone and will be unopposed by OPG until new osteocytes can be formed.

A limitation of our study is that we used gonadally intact male and female mice, whereas the majority of patients treated with denosumab are postmenopausal women. Nonetheless, the rebound resorption that we observed after discontinuation of denosumab in mice mimics that observed in postmenopausal women in that it exceeds pretreatment or control levels and is associated with loss of bone mass. In addition, the ability of denosumab to suppress bone resorption and increase bone mass in men is similar to that observed in postmenopausal women, suggesting that the mechanism of denosumab action is similar in both sexes (26).On the basis of these observations, we suggest our results in intact male and female mice are relevant to the rebound resorption that occurs in humans.

Our observations suggest that acceleration of the return of osteoblasts and newly formed osteocytes may blunt the magnitude of rebound resorption. Administration of antisclerostin Ab prior to denosumab discontinuation may be one approach to accomplish this. Consistent with this idea, recent studies have found that postmenopausal women treated with romosozumab for 12 months after 12 months of treatment with denosumab either had increased spinal BMD or maintained hip BMD (27–29). Markers of bone resorption increased after denosumab discontinuation, but it is not clear if romosozumab blunted the increase, because all patients were treated with romosozumab (27–29). In contrast, a case report of a single patient suggested that romosozumab was unable to block rebound resorption after discontinuation of denosumab (30). It should be noted that romosozumab was also unable to elevate a circulating marker of bone formation above baseline levels in this patient (30).

In summary, we have developed a murine model of rebound resorption and used it to identify some of the cellular and molecular changes that may set the stage for rebound resorption. This model should be useful to test potential interventions to blunt rebound resorption, such as romosozumab administration or tapering of the denosumab dose. In addition, because of the profound reduction in osteoclast and osteoblast number observed after denosumab administration, this model may also be useful to help identify the molecular mediators that couple bone formation to bone resorption and the osteoblast progenitors targeted by such factors.

## Methods

### Generation of hRANKL mice.

We used CRISPR-Cas–based gene editing to alter the coding sequence of the endogenous murine *Tnfsf11* gene in C57BL/6 mice. The nucleotide sequence encoding exon 5 was altered to create the following changes in 4 amino acids: S229D, V230L, P231A, and D233E. The amino acid number is based on the murine sequence. Gene editing was performed by microinjection of high-fidelity Cas9 protein (Sigma-Aldrich), sgRNA (Synthego), and single-stranded homology donor (Integrated DNA Technologies) into fertilized eggs from C57BL/6 mice. Founders were identified by PCR amplification and DNA-Seq of the modified region. Germline transmission of the modified allele was confirmed by DNA-Seq and positive mice were intercrossed to produce mice homozygous for the humanized allele. The following primers were used for genotyping: Rankl-inner-forward. 5′-GATCTAGCCACCGAGTATCTGCAGGTT-3′; Rankl-outer-reverse, 5′-ATTCAGAATTGCCCGACCAG-3′; product size, 121 bp for the humanized allele; Rankl-outer-forward, 5′-TCCTGTGCCTTTGTTTCTCTC-3′; Rankl-inner-reverse, 5′-CAGCTGAAGATAGTCTGTAGGTACGCT-3′; product size, 206 bp for the WT allele.

### Animal housing and care.

Mice were socially housed at 2–5 animals per cage using a blend of 0.25″ (0.6 cm) corncob bedding and white enrichment paper, both produced by Andersons Inc. The animal colony was specific-pathogen-free based on the Division of Laboratory Animal Medicine’s exclusion list. Mice were provided ad libitum water and an irradiated Purina diet of either 5V5M (for breeders) or 5V5R (for maintenance). The temperature range in the room was 68°F–79°F (20°C–26°C) with a set point of 71°F ± 2°F (22°C ± 2°C), and the humidity range was 30%–70%. The room was on a 12-hour light/12-hour dark cycle and the illumination was 364 lux measured 1 m from the floor.

### Denosumab administration.

Denosumab (Prolia; 60 mg/mL) was purchased from the University of Arkansas for Medical Sciences pharmacy, aliquoted into 0.5 mL sterile microtubes, and stored at 4°C protected from direct light. Mice were injected s.c. with either vehicle (saline) or denosumab at a dose of 5 or 10 mg/kg BW, in a total volume of 100 μL, once every 2 weeks for 3 or 4 doses or once a week for 3 weeks. These dosing regimens were based on work by others (5) and on our pilot studies.

### Serum assays.

Blood was collected by retro-orbital bleeding and allowed to clot at room temperature for 1 hour, after which the blood was centrifuged for 10 minutes at 1000*g* and the serum collected and stored in aliquots at –80°C. Kits for measuring TRAP5b (MouseTRAP ELISA), CTX-1 (RatLaps EIA), and P1NP (Rat/Mouse P1NP EIA) were purchased from Immunodiagnostic Systems, Inc., and used according to the manufacturer’s instructions.

### Quantification of bone mass.

We used a PIXImus densitometer (GE-Lunar Corporation) to measure BMD. The total body measurement encompassed the entire body excluding the calvarium, teeth, mandible, and the majority of the tail (with the exception of the first few caudal vertebrae). Isoflurane sedation was used during the 4 minute scan. Animal respiration and righting reflex were monitored during and after sedation. We used μCT to measure cortical and trabecular architecture of the femur. Bones were dissected, cleaned of soft tissues, fixed in 10% Millonig’s formalin containing 4% sucrose overnight, and dehydrated into 100% ethanol. Dehydrated bones were scanned using a model μCT40 scanner (Scanco Biomedical) to generate 3D voxel images (1024 × 1024 pixels). A Gaussian filter (σ = 0.8; support, 1) was used to reduce signal noise and a threshold of 220 was applied to all scans, at medium resolution (*E* = 55 kVp; *I* = 145 μA; integration time, 200 ms). All trabecular measurements were made by drawing contours every 10–20 slices and using voxel counting for bone volume per tissue volume and sphere-filling distance transformation indices. Calibration and quality control were performed weekly using 5 density standards, and spatial resolution was verified monthly using a tungsten-wire rod. Beam hardening correction was based on the calibration records. Nomenclature, symbols, and units used adhered to guidelines published by the American Society for Bone and Mineral Research (31).

### Histology.

Murine femurs, lumbar spine, and knee joints were processed for paraffin sectioning by first fixing in Millonig’s 10% buffered formalin for 24 hours. Bones were then decalcified in 14% EDTA for 1 week, after which they were dehydrated into 100% ethanol and embedded in paraffin for sectioning. Human cortical bone samples consisted of portions of the femoral neck obtained from discarded tissue after hip arthroplasty. The samples were obtained from 2 male patients (48 and 59 years old) and one female patient (73 years old), with no pathologies or medication use that could affect bone mass or architecture. Collection of human samples was approved by the University of Arkansas for Medical Sciences IRB (protocol 262666). Fragments of cortical bone were decalcified in 5% formic acid for 15–30 days and then embedded for paraffin sectioning as described above. For samples to be used for RNAscope, all aqueous solutions were prepared with diethyl pyrocarbonate–treated water. Murine vertebral bone for nondecalcified histology was fixed in Millonig’s 10% buffered formalin containing 4% sucrose, dehydrated into 100% ethanol, and then embedded in methyl methacrylate. For static and dynamic histomorphometry, 5 μm sections were used. Bones used for dynamic histomorphometry were obtained from mice that were injected with calcein i.p. (20 mg/kg BW) 7 and 3 days before tissue harvest. Staining for TRAPase was performed to identify osteoclasts in paraffin sections counterstained with hematoxylin or in plastic sections counterstained with toluidine blue. Histomorphometry was performed using an Olympus BX53 microscope and Osteomeasure software (OsteoMetrics, Inc.). Terminology used was that recommended by the American Society for Bone and Mineral Research (32).

### RNAscope.

All products were from Advanced Cell Diagnostics unless otherwise indicated. RNA ISH was performed using the RNAscope 2.5 HD detection reagent RED (catalog 322360) and Duplex (catalog 322500) kits and following the manufacturer’s instructions. In brief, 8 μm, paraffin-embedded bone sections were incubated at 60°C for 1 hour, deparaffinized, and pretreated with RNAscope Hydrogen Peroxide (catalog 322335) for 10–30 minutes at room temperature. Sections were then incubated in the custom pretreatment solution (catalog 300040) at 40°C for 10–45 minutes. The following probes were incubated on the sections for 2 hours at 40°C and detected with RNAscope 2.5 AMP 1-6 for RED kit and AMP1-10 for Duplex kit: murine *Acp5* (Mm-Acp5; catalog 465001), murine *Bglap* (Mm-Bglap; catalog 478941), murine *Tnfrsf11b* (Mm-Tnfrsf11b; catalog 488961), murine *Sost* (Mm-Sost; catalog 410031), human *BGLAP* (Hs-BGLAP; catalog 547451), human *TNFRSF11B* (Hs-TNFRSF11B-O1; catalog 430601), Hs-TNFRSF11B-C2; catalog 412291-C2), and human *SOST* (Hs-SOST; catalog 547451). Hybridization time for AMP 5, 6, 9, and 10 were modified for each probe. Signal was detected for 10 minutes at room temperature. Sections were counterstained with hematoxylin, dehydrated at 60°C for 20 minutes, and then mounted in VectaMount permanent mounting medium (Vector Laboratories).

Measurement of positively stained osteocyte distance from the bone surface was accomplished by scanning the slides at ×20 using an Aperio CS2 scanner (Leica Biosystems) and the distance to the nearest surface was measured using Aperio ImageScope software (Leica Biosystems).

Measurement of osteocyte signal intensity was performed using an algorithm developed in MATLAB to automatically segment histological images using thresholding techniques to extract the bone and the red signal within it. For each mouse, 80 nonoverlapping images of cancellous bone were obtained. For each field, a pair of images, 1 bright-field and the other fluorescent (excitation 575–625 nm), were obtained with the Olympus BX53 microscope and a ×40 objective at 59.176 nm per pixel resolution. Images were exported in TIFF to be used in MATLAB. To enhance bone surface in the fluorescence images, a top-hat filter of the red layer of the red-green-blue (RGB) image was used for a background estimation, followed by a subtraction operation to the red layer (33, 34). This step allowed removing bone marrow pixels outside of, or close to, the bone surface. Next, the well-known Otsu’s method was used to segment the bone by finding a threshold value that maximized interclass intensity variance and minimized intraclass intensity variance (35). This threshold was applied to the filtered images to obtain a binary mask of the bone.

To further clean the bone mask, morphological operations were applied to eliminate background pixels, fill area holes, and smooth edges. The bone mask was applied to the bright-field image to separate the bone from the background and to proceed with the segmentation of the red signal. The RGB masked-bone bright-field image was converted to the *Lab* (L: lightness; a: red/green value; and b: blue/yellow value) color space. Next, multilevel thresholding was determined for the *L*, *a*, and *b* layers and a composite segmentation mask was obtained. To extract the final red-signal mask, opening and closing morphological operations were computed to the mask to fill area holes and eliminate single pixels. The final red-signal mask was applied to the bright-field RGB image. The areas of the bone and red signal were calculated by counting the number of pixels in those regions. Conversion of pixels to meters was carried out using the conversion factor from the camera calibration setting. The percentage of the red signal was obtained by dividing the red signal area by the bone area.

As an alternative method to quantify changes in osteocyte *Tnfrsf11b* expression, *Tnfrsf11b^+^* or *Tnfrsf11b^–^* osteocytes were counted using an Olympus BX53 microscope with a ×40 objective and Osteomeasure software in the same vertebra used for the red signal area to bone area calculation described above.

### RNA isolation and gene expression analysis.

Total RNA was isolated from femoral or tibial cortical bone by homogenization in Trizol reagent (Life Technologies) according to the manufacturer’s instructions. Cortical bone was prepared by removing the periosteum with a scalpel and then flushing out the bone marrow. RNA was purified from freshly isolated bone marrow using the RNeasy Plus Mini kit (Qiagen). RNA quantity and 260:280 ratio were determined using a Nanodrop instrument (Thermo Fisher Scientific), and RNA integrity was verified by resolution on 0.8% agarose gels. We used 500 ng of RNA to synthesize cDNA using the High-Capacity cDNA Reverse Transcription Kit (Applied Biosystems) according to the manufacturer’s directions. Transcript abundance in the cDNA was measured by quantitative PCR using TaqMan Universal PCR Master Mix (Life Technologies) and TaqMan assays. PCR amplification and detection were carried out on an ABI StepOnePlus Real-Time PCR system (Applied Biosystems) as follows: a 2-minute holding stage at 50°C followed by a 10-minute initial denaturation at 95°C; 40 cycles of amplification including denaturation at 95°C for 15 seconds and annealing and extension at 60°C for 1 minute. The following TaqMan assays from Life Technologies were used: *Acp5* (catalog Mm00475698.m1), *Bglap* (for 5′-GCTGCGCTCTGTCTCTCTGA-3′, reverse, 5′-TGCTTGGACATGAAGGCTTTG-3′, probe, 5′-FAM-AAGCCCAGCGGCC-NFQ-3′), *Tnfsf11* (catalog Mm01313943.m1), *Tnfrsf11b* (catalog Mm00435452.m1), and the housekeeping gene ribosomal protein S2 (catalog Mm00475528). Gene expression was calculated using the comparative Ct method (36).

### In vitro osteoclast formation.

Total bone marrow cells were harvested from femurs by flushing with α-MEM complete medium containing 10% FBS and 1% penicillin–streptomycin–glutamine (Thermo Fisher Scientific). The nucleated bone marrow cells of each mouse were counted and cultured in a 12-well plate at a density of 1 × 10^5^ cells/well in 1 mL of α-MEM supplemented with 25 ng/mL recombinant Rankl (R&D Systems; catalog 462-TEC) and 25 ng/mL recombinant M-CSF (R&D Systems; catalog 416-ML-010) at 37°C and 5% CO_2_. The excess bone marrow cells from each mouse were immediately used to isolate total RNA for the detection of gene expression. The culture medium of each well was completely replaced with fresh medium on day 3, and cultured cells were fixed on day 5 with Millonig’s neutral buffered formalin (Leica; catalog 3800740) for 30 minutes and then stained using a TRAPase kit (Sigma; catalog 357A-1KT) according to the manufacturer’s instructions.

### 10X Genomics scRNA-Seq.

Myeloid lineage cells were isolated from hRANKL mice (13-month-old males) treated with 4 doses of vehicle or denosumab, and cells were harvested 2 weeks after the last dose, which was 8 weeks after the first dose. Cells were isolated from femoral and tibial bone fragments, after removing the periosteum and bone marrow, by digestion with Liberase (2 Wunsch units in 2 mL of HBSS) for 30 minutes at 37°C. The digestion was repeated once and the cells from both digests were pooled and incubated with an anti-Cd11b Ab conjugated to FITC (M1/70; eBioscience). Cd11b^+^ cells were isolated by FACS, after which their numbers and viability were quantified. Approximately 8000 cells/condition were encapsulated using a Chromium Controller (10X Genomics), and libraries were constructed using a Chromium Single Cell 3′ Reagent Kit (10X Genomics). The libraries were then sequenced using an Illumina NovaSeq 6000 machine to generate fastq files.

### Bioinformatic analysis of scRNA-Seq.

The fastq files were preprocessed using Cell Ranger software, version 6 (10X Genomics), to produce feature-barcode matrices. The alignments were performed using mouse reference genome mm10. The feature-barcode matrices were imported for further analysis in R suite software using the Seurat package, version 4.2.0 (37). The harmonization between samples was performed using a canonical correspondence analysis method based on the top 50 principal components and 6000 most variable features to minimize batch effect. The harmonized results were used for clustering using the Louvain algorithm with multilevel refinement and Uniform Manifold Approximation and Projection (UMAP) for dimension reduction. The gene-specific markers of individual clusters were identified using the function FindMarkersAll using the MAST algorithm for cell type identification (38). The expression level of the selected genes was plotted on the basis of the normalized expression value of relative count per 10,000. The scRNA-Seq data were deposited in the NCBI SRA database under Bioproject PRJNA896501 (https://dataview.ncbi.nlm.nih.gov/object/PRJNA896501?reviewer=hhpc16b01bnbnbtd6od170q90d).

### Reporter gene analysis.

Mice harboring the Dmp1-Cre or Sost-Cre transgenes, together with the Ai9 Cre reporter gene, have been described previously (39). Sections for reporter gene analysis were obtained from femurs by fixing overnight in 4% paraformaldehyde at 4°C followed by decalcification in 14% EDTA for 1 week, and then incubation in 30% sucrose for at least 1 day. Bones were then embedded in Cryo-Gel (Electron Microscopy Sciences) for frozen sectioning. We obtained 5 μm sections using a Leica cryostat with a tape-transfer system (Leica Microsystems; catalog LM3050S). Sections were mounted in SlowFade Diamond antifade medium with DAPI (Life Technologies) and then photographed using an Olympus BX53 microscope with a DAPI/FITC/Texas Red triple-filter cube. Td-tomato positive or negative osteocyte distance to the near surface and positive percentage were obtained through the software Aperio ImageScope (Leica Biosystems).

### Statistics.

With SAS, version 9.4, software, the outcomes of total BMD, serum PINP, serum TRAP, and serum CTX were analyzed by mixed-effects repeated measures models. Residuals of the models were examined for normality and equal variance. The model for serum TRAP and CTX did not meet these assumptions, so transformations of the raw data were examined. The square root transformation was applied and the resulting model did meet the assumptions of normality and equal variance. The models included random effects of animal and fixed effects of treatment, week, and the interaction of treatment and week. Pairwise comparisons of the 2 treatments at matching weeks were made with *t* tests and the resulting *P* values were adjusted by the FDR method. We also used SAS, version 9.4, software to analyze the distance of human *TNFRSF11B*^+^ and *SOST*^+^ osteocytes from the bone surface. To do this, 50 cells of each type for each of 3 study participants were randomly selected from all the cells measured for that participant. To meet assumptions of normality and constant variance, ranked data were used in place of the non-normally distributed raw data.

A nested ANOVA model was fit to the data with factors of cell type (*TNFRSF11B* or *SOST*) nested within the factor of individual participants. All other data were analyzed using Prism 8 (GraphPad Software). Two-tailed, unpaired Student’s *t* tests were used after determining that the data were normally distributed and exhibited equivalent variances. In the case of multiple comparisons, the Holm-Šídák method was used for correction. For data that were not normally distributed, the Mann-Whitney test was used.

### Study approval.

All animal procedures were reviewed and approved by IACUC of the University of Arkansas for Medical Sciences.

### Data availability.

Values for all data points shown in graphs and values behind reported means are provided in the Supporting Data Values file..

## Author contributions

QF and CAO designed and generated the hRANKL mice. QF, NCBG, HRP, IG, DEV, MP, JJG, and CAO performed experiments. EA, JBS, and CLB obtained human bone samples. IN performed bioinformatic analyses. JDT performed statistical analyses. QF, NCBG, and CAO analyzed results and wrote the manuscript. QF and NCBG are co–first authors. QF appears first in the author list because of his involvement in the initiation of the project. All authors edited and approved the final version of the manuscript.

## Supplementary Material

Supplemental data

Supporting data values

## Version 1 08/15/2023

In-Press Preview

## Version 2 09/22/2023

Electronic publication
