# Supplementary material for: Reduced osteoprotegerin expression by osteocytes may contribute to rebound resorption after denosumab discontinuation
Source: JCI Insight. 2023 Sep 22;8(18):e167790. doi: 10.1172/jci.insight.167790 (PMC10561722; doi:10.1172/jci.insight.167790)
Supplement: Supplemental data [file jciinsight-8-167790-s137.pdf]

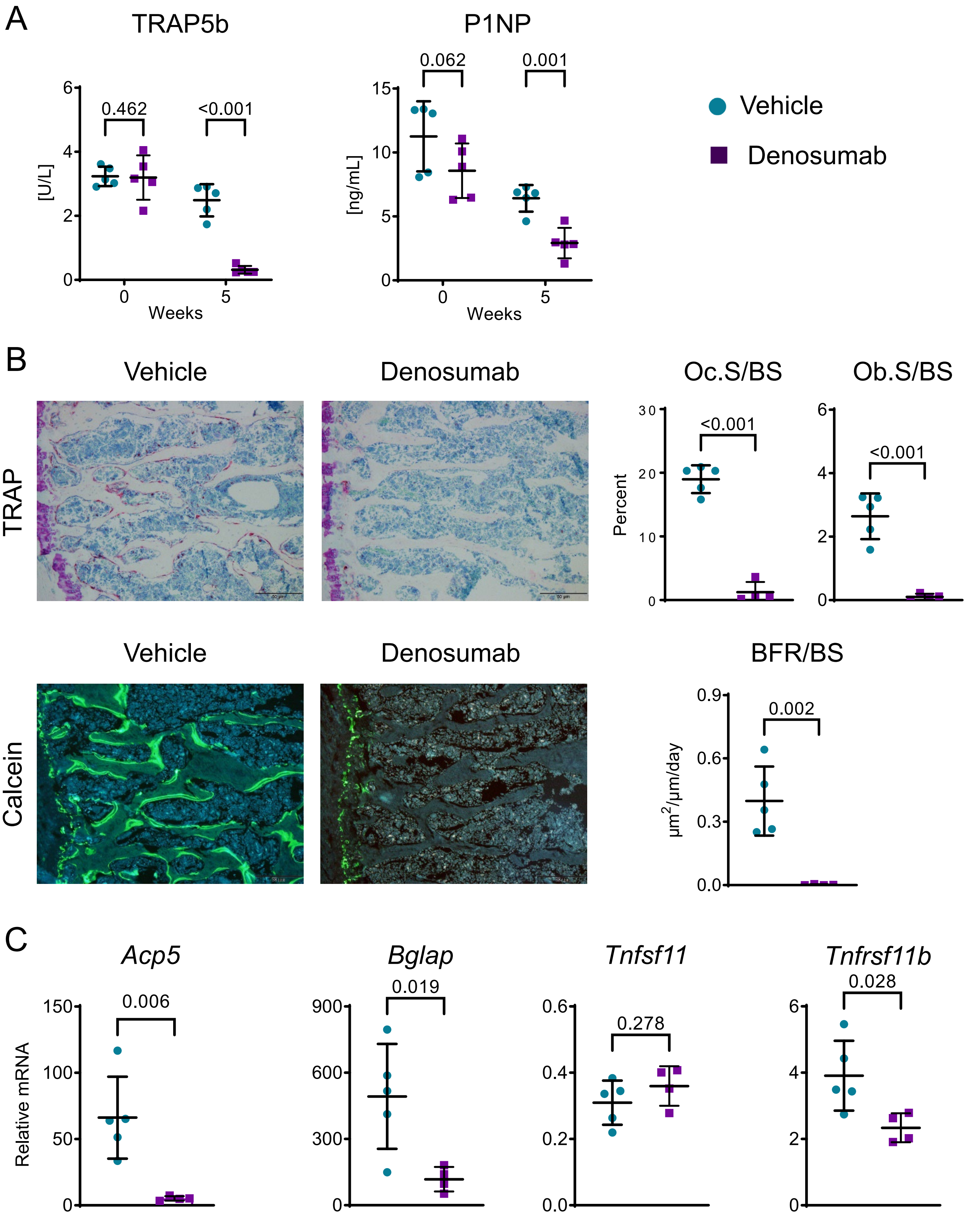
A
TRAP5b
P1NP
[U/L]
[ng/mL]
Weeks
0.462
<0.001
0.062
0.001
Vehicle
Denosumab
B
Vehicle
Denosumab
TRAP
Oc.S/BS
Ob.S/BS
Percent
<0.001
<0.001
Calcein
BFR/BS
μm²/μm/day
0.002
C
Acp5
Bglap
Tnfsf11
Tnfrsf11b
Relative mRNA
0.006
0.019
0.278
0.028

Figure S1

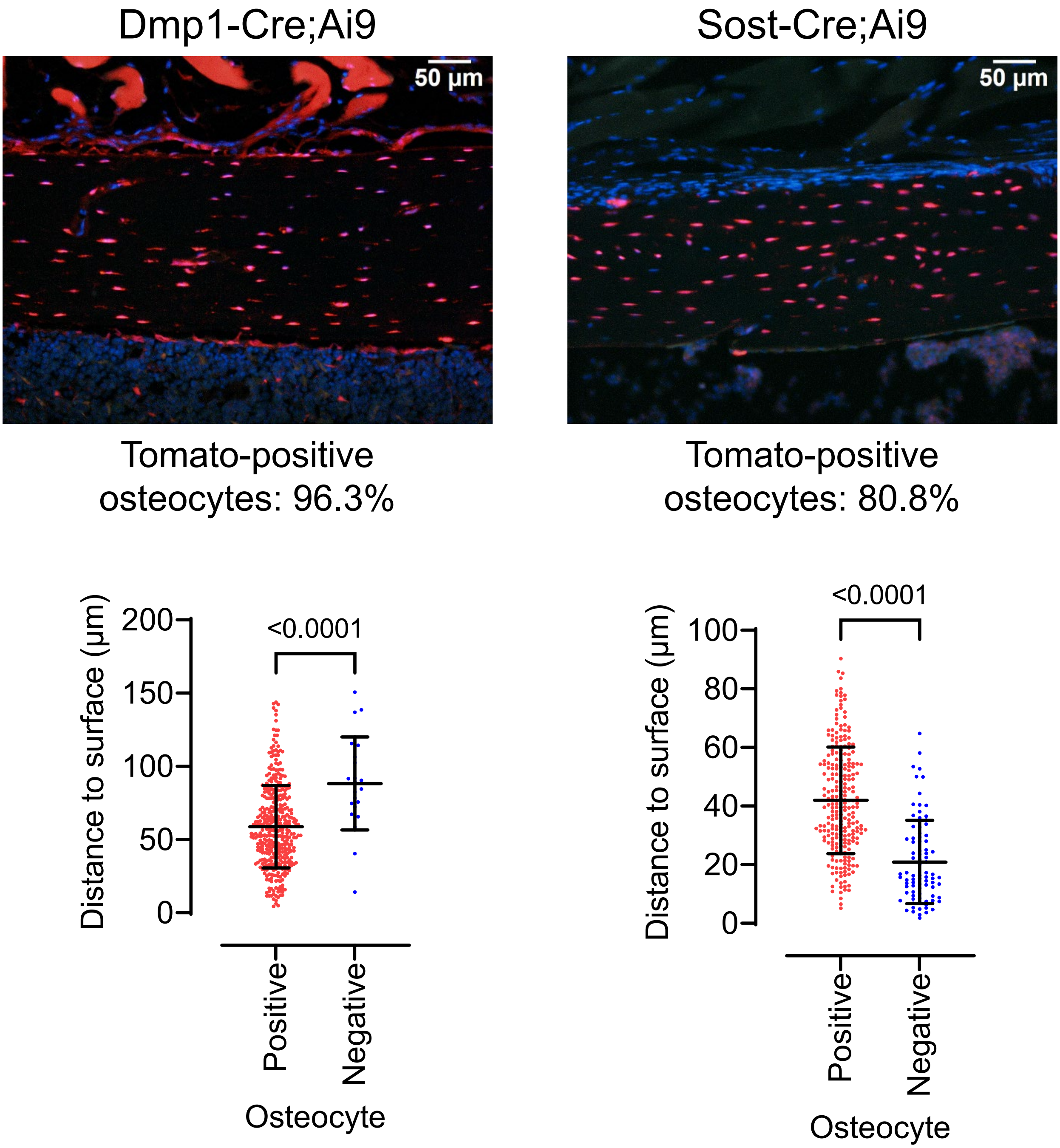
Dmp1-Cre;Ai9
Sost-Cre;Ai9
50 µm
50 µm
Tomato-positive osteocytes: 96.3%
Tomato-positive osteocytes: 80.8%
Distance to surface (µm)
200
150
100
50
0
<0.0001
Positive
Negative
Osteocyte
Distance to surface (µm)
100
80
60
40
20
0
<0.0001
Positive
Negative
Osteocyte

Figure S2

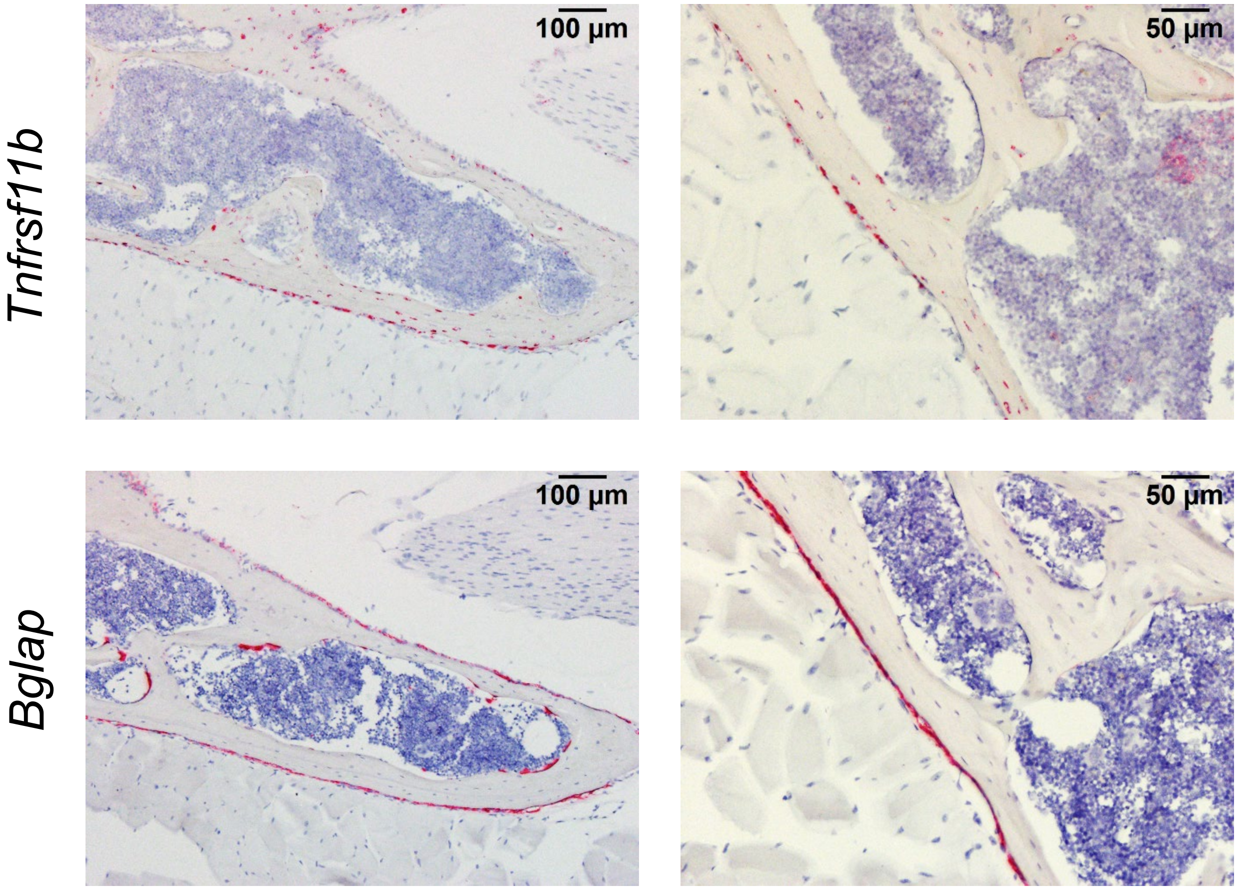

Figure S3

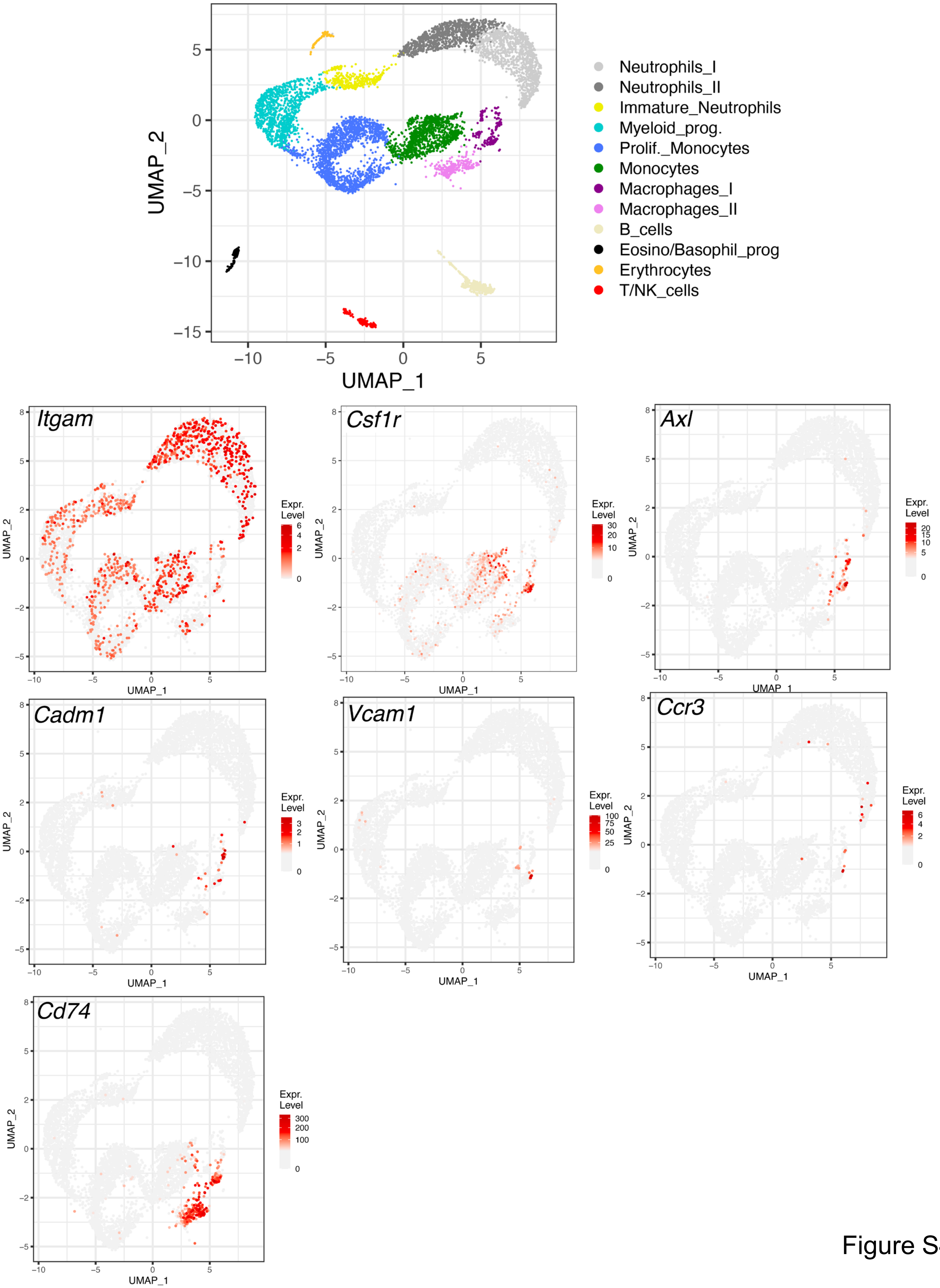
UMAP_1
UMAP_2
Neutrophils_I
Neutrophils_II
Immature_Neutrophils
Myeloid_prog.
Prolif._Monocytes
Monocytes
Macrophages_I
Macrophages_II
B_cells
Eosino/Basophil_prog
Erythrocytes
T/NK_cells
Itgam
Csf1r
Axl
Cadm1
Vcam1
Ccr3
Cd74
Expr. Level

Figure S4

Supplementary Figure Legends

Figure S1. Reproducibility of denosumab effects on bone remodeling. Nine-week-old male hRANKL mice were injected with vehicle or denosumab (5 mg/kg) once every 2 weeks for a total of 3 doses. Bones were harvested 1 week after the last dose. (**A**) Serum TRAP5b and P1NP measured 5 weeks after the first injection. n = 5 per group. (**B**) Vertebral histomorphometry. Top images are of vertebral sections stained for TRAPase and toluidene blue. Bottom images are of unstained vertebral sections viewed by epifluoresent microscopy. Oc.S/BS, osteoclast surface per bone surface; Ob.S/BS, osteoblast surface per bone surface; BFR/BS, bone formation rate per bone surface. Vehicle n = 5, denosumab n = 4. (**C**) Gene expression of the indicated genes using Taqman assays of RNA from cortical bone. Vehicle n = 5, denosumab n = 4. Bar and whiskers are the mean ± s.d. and *P* values are from 2-tailed unpaired *t* tests.

Figure S2. Sost-Cre does not target new osteocytes. Images of femoral bone sections viewed by epi-fluorescent microscopy from 4-month-old Dmp1-Cre;Ai9 mice and Sost-Cre;Ai9 mice. Numbers below the images indicate the percentage of osteocytes expressing the tdTomato reporter gene. A total of 793 osteocytes were counted for Dmp1-Cre and 625 for Sost-Cre. Graphs below the images show the distance of tomato-positive and tomato-negative osteocytes from the nearest femoral cortical surface. Bar and whiskers are the mean ± s.d. and the *P* value is from a Mann-Whitney test. Osteocytes for each transgene were measured in 2 mice.

Figure S3. Osteoblasts express *Tnfrsf11b*. In situ hybridization of vertebral bone from 5-month-old male mice using the indicated probes. The top panels show that some periosteal cells express *Tnfrsf11b* and that cells in the same region in serial sections also express *Bglap*, demonstrating that they are osteoblasts.

Figure S4. UMAP of genes expressed by osteomorphs in murine myeloid cells. The top panel shows the combined UMAP of cells from vehicle and denosumab-treated mice (6230 cells in total). UMAPs showing the expression patterns of osteomorph-related genes are shown below. Cd11b is encoded by *Itgam*.
